# Plant Diversity and Ethnoveterinary Practices of Ethiopia: A Systematic Review

**DOI:** 10.1155/2019/5276824

**Published:** 2019-01-06

**Authors:** Minyahel Tilahun, Melesse Etifu, Tesfaye Shewage

**Affiliations:** College of Agriculture and Natural resources, Wolkite University, Wolkite, Ethiopia

## Abstract

The systematic review was conducted on Ethnoveterinary Medicinal (EVM) plants from the two (integrated and pastoral) majorly known livestock production systems (LPS) of Ethiopia. A total of 48 documents pertinent to EVM significance were assessed from different sources using Google search engine and local university websites. Search outputs were screened using the developed inclusion criteria, and only 26 documents were selected. Descriptive analysis measures, Document Consensus Factor (DCF), and rank of the collected data were analysed using SPSS version 20 and Microsoft Excel. The result showed that females (33%), being below 40 years of age (27%), and educational level of above college (1%) healers participation was not significance. A total of 645 EVM plant species (from 133 families) were identified. Only 22 (16.54%) plant families were represented by one species. Leaf (47.8%) was the major plant part used to prepare remedies. The major administration route was oral route (58.2%). Blackleg 43 (0.188), diarrhea 25 (0.110), and wound 18 (0.079) were the most commonly treated livestock ailments. Solanaceae and Fabaceae were the frequently utilized EVM plant families in integrated and pastoral LPS, respectively.* Croton macrostachyus* (Bisana) and* Solanum incanum *(Embuay) were the most widely applied EVM plant species in integrated and pastoral LPS, respectively. Pastoral LPS were using higher number of specific EVM plants (DCF>0.5) compared to integrated LPS. Less than 40% (n< 10) of the collected documents were dealing with measurability and risk of toxicity, giving emphasis to indigenous plant and constraints of EVM plants use.

## 1. Introduction

In most African countries, traditional medicine has been bonded to people and animal health planning for centuries, and it has undergone a major revival for generations [1]. Ethiopia history of medicinal practice has long been recognized both in human and in livestock ailments treatment [2]. Although the livestock sector of Ethiopia has been estimated to contribute 19% of agricultural production by value, many hindering factors are presumed to be responsible for low livestock productivity and death of livestock [3]. Livestock ailment is the awful constraint which contributes to the death of about 8–10% of the cattle, 14–16% of the sheep, and 11–13% of the goat population [4].

Over 6,600 higher plant species are found in Ethiopia of which 22 are threatened [5]. About 30% of botanical medicinal preparations in Africa are probably effective [6]. EVM involves solid amalgamation of dynamic herbal known-how and ancestral experience [7]. It has particular importance in areas where modern veterinary services are absent, irregular, and/or expensive [8]. EVM has much to offer and can be a cheap and readily available alternative compared to costly imported drugs [9]. Traditional herbal knowledge has also an impact on the development of modern medicine [10].

Although every community has its own particular approach to health and disease even at the level of ethno-pathogenic perceptions of diseases and therapeutic behaviour, limited documents have been involved in multiethnolinguistic communities of Ethiopia. To overcome this, the very concept of systematic and scientific documentation of such knowledge is very important [11]. Hence, this document is designed to systematically review the EVM plants diversity and their use to treat livestock ailments in different LPS of Ethiopia.

## 2. Materials and Methods

### 2.1. Techniques of Data Collection

This systematic review was conducted between June 2017 and February 2018. The ecological zones used for this systematic review referred to agroclimatic zones classification of Ethiopia by Tessema Bekele [5]. Even though the agroclimatic zones are seventeen, only six, i.e., Alpine* Wurch* (>3700 masl),* Wurch* (>3200-3700 masl),* Dega* (>2300- 3200 masl), and* Weina Dega* (>1500-2300 masl), which reserve integrated LPS, and* Kolla* (500-1500 masl) and* Bereha* (< 500 masl) which reserve pastoral LPS were used to classify the agroclimatic zones into two (i.e., integrated and pastoral) broad LPS categories of Ethiopia based on the available and accessed document. Furthermore, this systematic review emphasized solely the importance of EVM on the two major LPS of Ethiopia.

### 2.2. Sources and Screening Criteria

A web-based systematic research literature search strategy was employed using keywords/ phrases “Ethno veterinary medicinal plant of Ethiopia”. Published and unpublished sources were accessed using Google search engine, local university websites, international scientific databases including “Pub Med”, “Science direct”, “Web of Science”, “Google scholar”, and “African Journals Online (AJOL)”.

Two stages were followed to screen out the search outputs from websites. First, the title and abstract of the identified documents were overviewed. Then, appropriate documents for the systematic review were downloaded/collected and critically inspected for fulfilling the inclusion criteria.

### 2.3. Inclusion Criteria and Exclusion Criteria

Although many authors/documents provided multiple years of data, this systematic review focused only most recent years (i.e., the year 2005 onwards) of EVM documents. This considered the transition of vegetation cover and availability of plant species on the study areas, and further experimental and empirical study on this issue might require use of availability plant families and species on the study areas. Availability of necessary detailed data (i.e., plant part use, plant habit, scientific name, source, etc.) which describe the botanical description of those EVM plants to treat livestock ailments was also taken as a filtering factor.

Review articles, historical documents, or experimental studies were excluded bearing in mind their less rendering nature of originality, scarcity of descriptive and analyzable data, and difficulty for recommendation. Documents which focus on human related traditional medicine, documents which lack information about the study areas, and documents which did not properly describe informant's involvement and scientific name of plants were the other exclusion criteria. Nevertheless, the excluded documents were used in discussion part of this systematic review. Ultimately, based on the specified inclusion criteria, a total of 26 documents, i.e., 10 from integrated and 16 from pastoral LPS, were selected and used for this systematic review.

### 2.4. Data Retrieval

Family name of specific plant and misspelled scientific names were retrieved from Natural Database for Africa (NDA), Version I 2.0 [37]. In addition, documents which lack specific geographic locations/localities/districts information were retrieved through direct web (Google) searching.

### 2.5. Documents Consensus Factor (DCF)

The level of homogeneity within information provided by different informants/documents was calculated by the Informants' Consensus Factor (ICF) [38]. However, this systematic review modified the concept of ICF to Documents Consensus Factor (DCF) considering selected documents as an informant use of ICF, with the rest of the formula left as it is.

(1) $$\begin{array}{c} ICF=\frac{Nuc-Ns}{Nuc-1}\text{ Adapted to }DCF=\frac{Nur-Nt}{Nur-1} \end{array}$$

where Nur is number of use reports from documents for a particular plant use category and Nt is number of plant taxa or species used to treat livestock ailments from all the included documents. DCF value range was between 0 and 1, where ‘1' indicates the highest level of document consent and ‘0' indicates the lowest document consent. Generally, DCF value of above 0.5 indicates majority of the documents agree up on the specific plants use in treating livestock ailment.

### 2.6. Data Analysis

This systematic review employed descriptive and explanatory analysis. Mean and Standard Error of Mean (SEM) were performed with SPSS version 20.0, and Microsoft Excel was used to compare the DCF values of specific plant species which are used to treat livestock ailments among the documents, and to calculate indexes and ranks of livestock ailments treated by EVM plants on different LPS of Ethiopia.

## 3. Results and Discussion

### 3.1. Characteristics of the Reviewed Documents on EVM Use

Table 1 presents characteristics of the documents used in the systematic review. The documents used for this systematic review were characterized based on the developed inclusion criteria that only focused on EVM knowledge. One can predict the practice of EVM based on the number of livestock and the vegetation cover of specific area. Almost all ecological zones, ethnic groups and communities have been identified in trusting EVM without the limitations of the availability and accessibility of infrastructure. The systematic review revealed that 645 different EVM plant species from 133 families were identified from the included documents.

The systematic review showed that, of the informant and healers, only 33 % were females. This result is in line with [39] which stated the younger generation in Ethiopia is increasingly losing interest in learning about the medicinal herbs. The educational levels of all the informants or healers of the pastoral LPS were below college. The systematic review revealed that measure of reliability 13(43.3%), measurability 3(10%), toxicity risk assessment 1 (3.33%), and stressing constraints 4 (13.3%) were not given due stress on the included documents. In pastoral LPS, the proportion of plant families which were represented by more than one plant species 61 (67.8%) was higher than the integrated LPS 53 (45%). In contrast to this systematic review, [20] reports that only 31% of integrated LPS plant families are represented by more than one plant species. Respondent's or healer's knowledge of identifying the EVM botanical families and their corresponding species might bring variability in the number of identified plant species.

### 3.2. EVM Sources and Plant Habits

Table 2 describes the EVM plants sources and habits. Source of EVM plants for both LPS were majorly from wild sources (80.57±2.25). The systematic review revealed that market was not used as source of EVM plant for pastoral LPS. Herbs both from integrated and from pastoral LPS showed higher average proportion of 32.05±10.24 and 31.19±23.52, respectively. Pastoral LPS used lower average proportion (18.92±15.93) of trees as compared to integrated LPS (31.82±11.03). This might be due to plant reservation capacity of specific ecologies. Some EVM plants are available only in certain seasons of the year. This might often hinder the application of traditional medicine. Moreover, some of the preparations use mixtures of plants which are difficult to find at specific season [40].

### 3.3. Routes of EVM Administration

Table 3 presents EVM administration routes in integrated and pastoral LPS of Ethiopia. Oral route of administration (50.11±21.60) was the major route in both LPS. Topical and nasal routes were the second and third routes of administration in both LPS and at country level.

The proportion of dermal (8.11± 2.8) and nasal (13.39± 3.8) routes of administrations in pastoral LPS were much higher than integrated (2.93± 2.07; 5.74± 1.84) LPS. This might be due to the fact that prevalence rate of dermal and respiratory ailments in pastoral LPS is higher than other livestock ailments. The result of this systematic review is in line with [13–16, 18, 30].

### 3.4. Plant Part Used for Treatment

**Figure 1:**
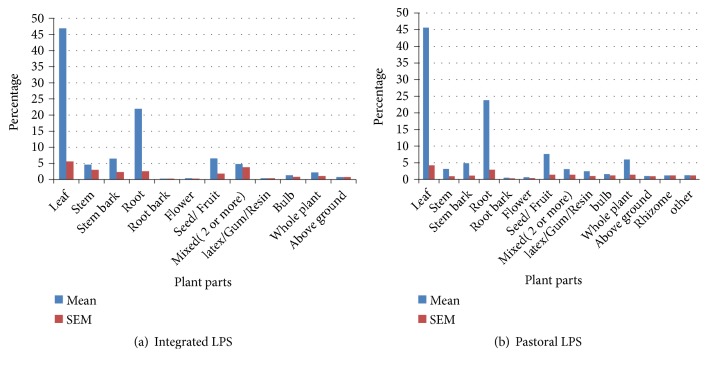
(a) Integrated and (b) pastoral LPS plant parts used to treat livestock ailments.

Different plant parts used in treating livestock ailments are described in Figures 1(a) and 1(b). Leaf was the major plant part used to treat livestock ailments both in integrated (46.89%) and pastoral (45.59%) LPS of Ethiopia. The second and third majorly used plant parts both in integrated (21.94%; 6.53%) and pastoral (23.81%; 7.66%) LPS were root and seeds/fruit, respectively. The finding of this systematic review is in line with [13, 16].

### 3.5. Major Livestock Ailments Treated and DCF of EVM from the Selected Documents

Frequently treated livestock ailments and DCF value of major EVM plant species are presented in Table 4. Black leg was indexed first 43 (0.188) among the different documented ailments. Tree species like* Justice schimperiana*,* Allium sativum*, and* Lapidum sativum* were the frequently documented plant species used to treat blackleg. Other livestock ailments, i.e., diarrhea 25 (0.110), wound 18 (0.079), and bloat 17 (0.073), were ranked second, third, and fourth, respectively. Most ailments treated in pastoral LPS showed higher DCF value (i.e., DCF> 0.5) than integrated LPS for similar ailments. This might be due to the fact that availability of different EVM plants in integrated LPS could let healers/respondents depend on various EVM plants rather than specific EVM plant. This result is supported by the works [9, 12, 13, 16, 20, 22, 34] which indicates different DCF value of livestock ailment from the selected documents reveal that less than six plants are specifically used to treat specific livestock ailment from integrated LPS. The majorly treated livestock ailments of this study were supported by number of documents [19, 20, 29, 34]. However, this result is in contrast with [18, 27] which indicate the majorly treated livestock ailments are diarrhea and wound rather than black leg. The in agreement might be due to the prevalence rate of most livestock ailments from different agroecology oblige healers and respondents to focus on frequently occurring ailment with long years of experience in treatment.

### 3.6. Frequently Utilized EVM Plants

Table 5 presents the frequently utilized EVM plants in pastoral and integrated LPS of Ethiopia. The frequently utilized EVM plant family was Fabaceae 45 (8.11%) and Solanaceae 44 (7.94%) for pastoral and integrated LPS, respectively. Fabaceae 72(11.16%) was ranked first at country level.* Croton macrostachyus *(*Bisana*) 15(21.43%) was the frequently utilized plant species to treat livestock ailments of the country. However, its contribution was higher in integrated 7(70%) than pastoral 8(50%) LPS of Ethiopia.* Solanum incanum *(*Embuay*) 9(75%) was the principal EVM plant species in pastoral LPS of Ethiopia. The result of this systematic review is supported by [14, 16, 30]. Other studies [13, 16, 19, 20, 22, 26, 27] report that the frequently utilized plant families and species are in contrast to the finding of this systematic review. Agroecological factors can determine the type and abundance of EVM plants that can grow in an area [5]. Moreover, the utilization frequency of those effective EVM plant species might coincide with the presence of bioactive ingredients against livestock ailments. The frequency of using such EVM plant families and species might be correlated to the prevalence rate of some livestock ailments such as ticks and flies in pastoral LPs demand specific EVM plant species like* Calpurnia aurea* (Hits awuts) (Table 4). A common Amharic proverb* ‘Gizawa eyale edejishi lije motebign tiyalesh'* acknowledges the ubiquitous significance of* Withania somnifera* and its astonishing uses against evil eye and sudden death. Furthermore, the proverb also indicates communities' faith on medicinal plants healing power even to the worst extreme of life which is death.

## 4. Conclusion

The present systematic review revealed that Ethiopia has rich EVM plant diversity, i.e., 645 different plant species from 133 families. Among the EVM plant families, Solanacea and Fabaceae are the major EVM plant families in integrated and pastoral LPS, respectively.* Croton macrostachyus *(Bisana) and* Solanum incanum *(Embuay) are the frequently utilized EVM plant species in integrated and pastoral LPS of Ethiopia, respectively. Black leg (*Aba gorba*), diarrhea (*Tekimat*), wound (*Kusil*), and bloat (*Yehod meketet*) are the four mostly treated livestock ailments. Higher number of livestock ailments from pastoral LPS has a DCF value of greater than 0.5 as compared to integrated LPS. Women and younger generation contribution and involvement in the preparation and knowledge development of ethnic medicine are very insignificant. Moreover, the majority of the documents lack information about herbal toxic effect, dosage, measurability and conservation. Revealing the appropriate dosage in divergent preparation and use patterns of herbal remedies among multiethnolinguistic communities, as well as associated toxicity risks and countermeasures, generally demand deeper and exhaustive investigations. Therefore, sustainable development and exploitation strategy which focus on protecting the endangered medicinal plants necessitate coordinated multidisciplinary research programs that give due credit to create responsible traditional practitioners and commercial investors.

## Figures and Tables

**Table 1:** Summary statistics of the documents used in the systematic review.

Measured variables		Criterion	LPS n (%)		Total N (%)
			Integrated	Pastoral	
			(n=10)	(n=16)	
No of reviews(N=48) and article type		Published	10(100)	14(87.5)	28(93.3)
		Unpublished	0(0)	2(12.5)	2(6.66)

Diversity of plant family(N=133)		Identified species	373	555	645
		one species	39(45.1)	29(32.22)	68(37.36)
		> one species	53(44.9)	61(67.78)	114(62.64)

Number of participant (*n=19*)	Gender	Male	634(73.21)	585(75.57)	1219(66.94)
		Female	232(26.79)	370(17.83)	602(33.06)

Representativeness	Age	< 40 years old	202(36.27)	76(19)	246(27.21)
		> 40 years old	355(63.73)	324(81)	658(72.79)
	Literacy level/Education (*n=9*)	Illiterate	183(55.30)	110(37.07)	293(42.19)
		read and write	19(8.49)	34(26.33)	53(17.41)
		primary School	304(30.08)	149(32.93)	453(31.51)
		Secondary School	10(4.44)	6(2.88)	16(3.66)
		College and Above	2(1.74)	0	2(0.87)

Coverage of agricultural zones	More than three		9(90)	0	9(34.62)
	Two		1(10)	7(43.75)	8(30.77)
	One		0	9(56.25)	9(34.62)

Studies reliability	Used same language as informants		5(35.7)	8(50)	13(43.3)

Measurability	Specifying livestock No.		1(7.14)	2(12.5)	3(10)

Risk of toxicity			0	1(6.25)	1(3.8)

Stressing on endemic plant species			0	2(12.5)	2(7.7)

Stating the constraints			2	2(12.5)	4(15.4)

Information on veterinary coverage			0	0	0

References used for analyzing the EVM contribution in Integrated and pastoral LPS			[12–21]	[9, 22–36]	

Note that numbers written under square brackets are references/documents used for the analysis of the systematic review.

**Table 2:** Sources and habits of EVM plants.

Sources and plant habits^*∗*^	LPS (mean± SEM)		Total (N=26)
	Integrated (*n*=10)	Pastoral (*n*=16)	
Wild	80.57± 0.71	79.71±3.55	79.97± 2.42
Cultivated	13.79± 1.68	16.82± 2.05	15.89± 1.51
Market	1.01± 0.64	0.00±0	0.31± 0.22
Mixed sources	4.60± 2.09	3.52± 2.45	3.85± 1.81

Shrub^*∗*^	27.31± 4.59	20.48± 4.6	26.59± 3.02
Herbs^*∗*^	32.05± 3.24	31.19± 5.88	36.47± 3.1
Trees^*∗*^	31.82± 3.49	18.92± 3.98	27.34± 2.56
Climbers^*∗*^	7.93± 2.19	6.03± 1.3	7.46± 1.11

Note that mixed sources represent any of i.e. wild, cultivated and market can be the sources of EVM plant (only for integrated LPS); plant habit means ability of plants to adapt to its evolving environment.

**Table 3:** EVM administration routes in integrated and pastoral LPS of Ethiopia.

Routes of administration	LPS (mean± SEM)		Total (N=26)
	Integrated (n=10)	Pastoral (n=16)	
Oral	60.14±4..31	46.77±5.31	50.11± 4.23
Topical	22.58±3.78	22.01±5.76	21.96± 3.79
Dermal	2.93± 2.07	8.11± 2.8	5.81± 0.56
Fumigation	1.44± 1.01	0.87± 0.65	1.03±2.5
Nasal	5.74± 1.84	13.39± 3.8	9.93± 0.86
Ocular	1.70± 1.06	2.51± 0.93	2.48± 0.59
Aerosol	0.44± 0.44	0.25± 0.24	0.31±0.22
Auricular	1.40± 1.40	1.87± 1.55	1.62±1.05

**Table 4:** Frequently treated diseases and DCF of major livestock ailments.

Livestock disease	Vernacular Name	*N*(Index)	Rank	*Plant species* (Amharic) (N=645)	LPS				Total
					Integrated		Pastoral		
					*Nur(Nt)*	DCF	*Nur(Nt)*	DCF	DCF
Black leg	*Aba gorba*	43(0.188)	1^st^	*Justicia schimperiana *(Sensel), *Allium sativum *(Nech Shinkurt), *Lepidium sativum *(Feto), *Momordica foetida *(Yekura hareg), *Croton macrostachyus *(Bisana),** ***Solanum incanum* (Embuay),** ***Withania somnifera *(Gizewa)	5.25(6)	0.15	3.53(14)	0.81	0.84
Diarrhea	*Tekimat*	25(0.110)	2^nd^	*Ocimum *lamifolium (Damakese), *Olea europaea* sub sp. (Weyira), *Zingiber officinale *(Zingible),	4(7)	0.5	2.41(15)	0.90	0.91
Wound	*Kusil*	18(0.079)	3^rd^	Caricapapaya (*Papaya*), *Prunus Africana *(Gebrch)	4(8)	0.57	3.88(13)	0.76	0.85
Bloat	*Yehod meketet*	17(0.073)	4^th^	*Lepidium sativum*;* Croton macrostachyus*;* Zingiber officinale*	2.88(7)	0.69	1.12(9)	0.98	0.95
Internal Parasite	*Yewist Tigegna*	14(0.061)	5^th^	*Vernonia amygdalina* (Gerawa);** ***Hageniaabyssinica* (Kosso*);*	3.75(5)	0.31	1.76(10)	0.92	0.9
External Parasite	*Yewich Tigegna*	13(0.057)	6^th^	*Calpurniaaurea* (Hits awuts)	3.50(7)	0.58	2.47(15)	0.89	0.91
Mastitis	*Yetut beshita*	12(0.052)	7^th^	*Achayrentes aspera *(Telenj); *Clorodandrum myricoidos *(Michi-ashit); *Brucea antidysenterica *(Weyinos/Yekola Wanza)	2.50(5)	0.63	1(7)	1	0.96

Note that plant species name under prentices represent Amharic language.

**Table 5:** Most frequently utilized EVM plant families and species used to treat livestock ailments.

Plant Family (N=133)	LPS				Plant Species (N=645)	Vernacular name	LPS n (%)		Total N (%)	Rank
	Integrated (n=373)		Pastoral (n=555)				Integrated	Pastoral		
	*n* (%)	Rank	*n* (%)	Rank						
Asteraceae	33(8.85)	2	36(6.5)	3	*Vernonia amygdalina*	Girawa(Am)/ Gara/ Hecho/ Ebichaa/ Hecho/ Eebicha/ Calyocad	4(40)	8(50)	12 (46.15)	3

Euphorbiaceae	24(6.4)	4	40(7.21)	2	*Croton macrostachyus*	Bisana(Am)/Anka/ Masencho /Tmbaho/Makkanisa/ Bakkanniisa/Mekanisa/ Ulee fooni /Bekenisa	7(70)	8(50)	15 (57.69)	1
					*Ricinus communis*	Qobo (Am)/Key qobo/ Gulee/ Qobboo diimaa/ Gulo	5(50)	4(25)	9(34.6)	6

Solanaceae	44(7.94)	1	26(6.97)	4	*Solanum incanum*	Embuay(Am)/ Buluwa / Roriko/ kolodo'ita,wakere ku'us/ Hiddi/ Garint/ Hiddi/ Waniiye/ Angule/ Hidi	3(30)	9(75)	12 (46.15)	3
					*Withania somnifera*	Gizawa(Am)/Edigagga/Mixmixa/kokerabito,ubalto/Agol/Gizaawwaa/ Guryofan/ Gizewa/Tiro/Hidi bude	7(70)	4(25)	11 (42.31)	4
					*Nicotiana tabacum*	Tembaho(Am)/ Tamboo/ Bangiso/ Tamboo/Jiic / Tobako/ Tumbo/ Tambuk	6(60)	8(50)	14 (53.84)	2

Acanthaceae	6(1.61)	8	13(2.34)	8	*Justicia schimperiana*	Sensel(Am)/ werabikela/ Shimeja/ Dhummugaa	2(20)	5(31.25)	7 (26.92)	8

Fabaceae	27(7.24)	3	45(8.11)	1	*Calpurnia aurea*	Hitsawts(Am) /Mello/ Hsaws/Kaino/Ceekkataa/Dhekat/Digita/Cheketa/Hitsawuts	4(40)	6(37.5)	10 (38.46)	5

Phytolacceae	5(1.34)	9	4(1.07)	11	*Phytolacca dodecandra*	Indod(Am)/Haranjicho/Shifiti/Andoodee/Handoodee/ Arenji/irtsets	6(60)	4(25)	10 (38.46)	5

Lamiaceae	19(5.09)	5	26(4.68)	5	Most of the species frequency is below two					

Caparidaceae	1(0.27)		18(3.24)	6	Most of the species frequency is below three (i) *Cadaba farinosa Forssk* (ii) *Cadaba rotundifolia Forssk*	Dinibayto /Shifoweyin Cadaba rotundifolia Forssk				

Cucurbitaceae	16(4.29)	6	18(3.24)	6	Most of the species frequency is below two					

Rutaceae	10(2.68)	7	12(2.16)	9	*Ruta chalepensis*	Tenadam(Am)/Xalataam/Shanfae/Chena adam/Telatam/Taltan/ Tsetala	3(30)	6(37.25)	9(34.6)	6

Sapindaceae	4(0.80)	11	5(0.90)	10	*Dodonea angustifolia*	Kitkita(Am)/Senkara/ Dhiddecha/Tahises /Itacha/ Eticha/Tahsus	3(30)	6(37.25)	9(34.6)	6

Amaranthaceae	4(1.07)	10	15(2.70)	7	*Achayrentes aspera*	Telenj(Am)/Darguu/Mechelo/Mechalo/Maxxannee/ Begegechoo	3(30)	4(25)	7(26.92)	8

Zingibetaceae	3(0.80)	12	5(0.90)	10	*Zingiber officinale*	Zingibil(Am)Zinjibl/Ginger/Jijimbila /Jinjible/Singibill/	5(50)	3(18.75)	8(30.77)	7

Note that different EVM plant species vernacular names are from different ethnic groups of Ethiopia. “Am” represents Ethiopian national language which is Amharic language; “n” represents the number of plant species on each LPS.
